# Development of a prognostic model for sepsis based on gut microbiota-associated genes and identification of potential targets

**DOI:** 10.3389/fmed.2026.1766359

**Published:** 2026-03-05

**Authors:** Fangqiong Li, Minrong Xu, Huiqin Xiao, Ping Hu, Wei Zhang

**Affiliations:** 1 Department of Clinical Laboratory, Tongde Hospital of Zhejiang Province Affiliated to Zhejiang Chinese Medical University, Hangzhou, China; 2 Department of Critical Care Medicine, Tongde Hospital of Zhejiang Province, Hangzhou, China

**Keywords:** gut microbiota, molecular docking, prognostic signature, retinoic acid receptor-related orphan receptor alpha, sepsis, single-cell RNA sequencing

## Abstract

**Background:**

Gut microbiota dysbiosis drives sepsis progression by impairing intestinal barrier function and exacerbating systemic inflammation, but the microbiota-host-immune interaction mechanisms remain unclear.

**Methods:**

We integrated transcriptomic and single-cell RNA sequencing (scRNA-seq) data from the Gene Expression Omnibus (GEO) database. Differentially expressed genes (DEGs) between sepsis patients and healthy controls were identified in GSE154918, then intersected with 248 gut microbiota-related genes from the GutMGene database to obtain candidate genes. A prognostic model named GMGscore was constructed via LASSO-Cox regression in GSE65682 and validated in GSE95233. Area under the curve (AUC) was used to evaluate the model performance. The expression of gut microbiota-related genes was validated in peripheral blood samples obtained from patients with sepsis through RT-qPCR. Furthermore, scRNA-seq data (GSE167363) was used to determine the cellular localization of key genes. Molecular docking predicted interactions between gut microbiota metabolites and the key target.

**Results:**

We identified 34 gut microbiota-related DEGs, which were enriched in pathways like inflammatory bowel disease and IL-17 signaling. The GMGscore, based on 6 genes (CYP1A2, FFAR2, IL4R, MUC1, RORA, ASPM), showed excellent prognostic performance (AUC = 0.903 in training set; AUC = 0.901 in validation set). High GMGscore correlated with poor survival, upregulated neutrophil degranulation and reduced neutrophils. RORA was identified as a key gut microbiota-related target, which was consistently downregulated in sepsis with the highest diagnostic AUC across datasets, mainly expressed in effector T cells and NK cells, and positively correlated with CD8 + T cell/NK cell infiltration (*R* = 0.419 and 0.352, respectively). Virtual knockout of RORA downregulated cytotoxic genes. Molecular docking showed stable binding of RORA with *Collinsella*-derived metabolites (Citric acid, Sedoheptulose, and Tricarballylic acid).

**Conclusion:**

The GMGscore is a robust prognostic tool for sepsis. RORA, targeted by gut microbiota metabolites, may regulate immune balance via effector T cells and NK cells. These findings advance understanding of gut microbiota-sepsis crosstalk and provide new avenues for precise prognosis and targeted therapy.

## Highlights

- Multi-omics data were integrated to construct a prognostic model (GMGscore) based on 6 core genes (CYP1A2, FFAR2, IL4R, MUC1, RORA, ASPM).
- The GMGscore exhibited excellent performance in both the training set (AUC = 0.903) and validation set (AUC = 0.901).
- RORA was identified as a key target for gut microbiota intervention.
- Molecular docking further revealed that Collinsella-derived metabolites (citric acid, sedoheptulose, tricarballylic acid) could bind stably to RORA.

## Introduction

Sepsis is one of the leading causes of death in intensive care units (ICUs) worldwide, characterized by an excessive inflammatory response and immune suppression imbalance triggered by infection (1). According to epidemiological reports, sepsis has been recognized as a global health burden since 2017 due to its high incidence and mortality rates, with an estimated global incidence of approximately 49 million cases and a mortality rate as high as 20–30% (2). The annual healthcare costs associated with sepsis are estimated to be $24 billion (3, 4). Although the Surviving Sepsis Campaign (SSC) guidelines recommend early anti-infective therapy and fluid resuscitation (5, 6), sepsis patients exhibit significant individual variability. Conventional prognostic markers, such as procalcitonin and C-reactive protein, lack sufficient specificity, making it difficult to achieve accurate risk stratification (7). Therefore, the development of prognostic models and therapeutic strategies based on molecular mechanisms is a critical need for reducing the mortality rate of sepsis (8–11).

Dysbiosis of the gut microbiota is one of the core drivers of the pathological progression of sepsis. Under septic conditions, impairment of the intestinal barrier function leads to bacterial translocation and the entry of harmful metabolites into the bloodstream, which exacerbates the systemic inflammatory response and forms a vicious cycle (9, 12–15). Previous studies have confirmed that the composition and metabolites of the gut microbiota may affect an individual’s immune phenotype and the prognosis of sepsis (16–18). Thus, the development of gut microbiota-associated biomarkers can provide a more comprehensive perspective for the prognostic evaluation of sepsis (19). In addition, gut microbiota-related interventions have been shown to improve intestinal barrier function and survival rates in septic mice (16, 20, 21). However, the interaction mechanisms among the gut microbiota, host genes, and immune system in sepsis, as well as the key regulatory targets, remain unclear, which limits the clinical translation of precise interventions.

In this study, through multi-omics integrated analysis, we aim to find out the core prognostic genes associated with the gut microbiota in sepsis, construct a quantifiable prognostic score, designated as GMGscore, and identify potential targets that can be intervened via gut microbiota modulation and explore their underlying molecular mechanisms.

## Materials and methods

**Figure 1:**
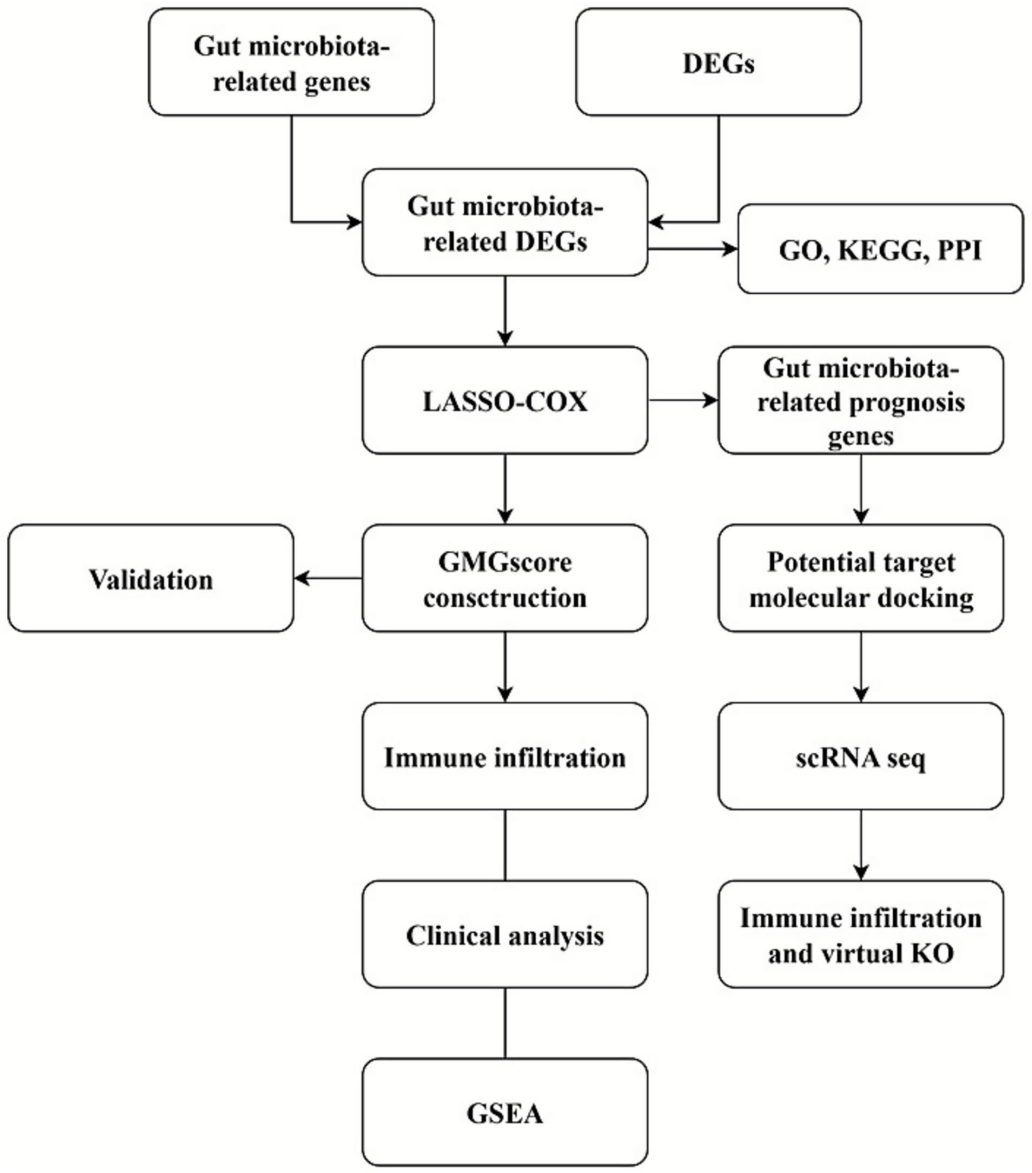
Flow chart of this current study.

The complete flow chart of this study is shown in Figure 1.

### Data sources

All datasets used in this study were retrieved from the Gene Expression Omnibus (GEO) database (https://www.ncbi.nlm.nih.gov/geo/). Only peripheral blood samples (whole blood or peripheral blood mononuclear cells) were included. The detailed information of all datasets is provided in Supplementary Table S1.

The GutMGene database (http://bio-computing.hrbmu.edu.cn/gutmgene/#/home) is a comprehensive repository that collects genes confirmed to interact with gut microbiota and their metabolites through peer-reviewed studies (22). We retrieved all genes associated with gut microbiota and their metabolites from the GutMGene database, resulting in a total of 248 genes with detailed information available in Supplementary Table S2.

### Data preprocessing

Data normalization and quality control were performed using R software (Version 4.3.1): Low-quality cells were filtered out using the “Seurat” package (23) with the following criteria: mitochondrial gene content >10% and unique molecular identifier (UMI) count <200. The “NormalizeData” and “FindVariableFeatures” functions in Seurat were applied to normalize the count data and identify highly variable genes, respectively. Cell clusters were visualized via Uniform Manifold Approximation and Projection (UMAP) with a resolution of 1.0.

Marker genes were detected using the “FindAllMarkers” function in Seurat based on the Wilcoxon rank-sum test. Only markers associated with clusters that met the threshold of log2 fold change > 0.25 were retained. After clustering, cells were reclassified into subpopulations, and the identity of each subpopulation was determined based on the similarity of gene expression patterns. Cell type annotation was conducted by combining the “SingleR” package (24) and the CellMarker database (http://biocc.hrbmu.edu.cn/CellMarker/).

### Screening of candidate genes

Differentially expressed genes (DEGs) between sepsis patients and healthy controls in GSE154928 was analyzed using the “DESeq2” package (25). DEGs were screened under the criteria of |log2 fold change| > 1.5 and adjusted *p*-value < 0.05. Candidate genes associated with the gut microbiota were obtained by taking the intersection of “DEGs” and “gut microbiota-associated genes”.

### Functional and pathway enrichment analyses, and construction of protein–protein interaction (PPI) network

Gene Ontology (GO) analysis is a commonly used method to investigate gene expression based on cellular functions or localization, typically conducted at three levels: Biological Process (BP), Molecular Function (MF), and Cellular Component (CC). The Kyoto Encyclopedia of Genes and Genomes (KEGG) is a database that stores information on genomes, biological pathways, diseases, and drugs. ClusterProfiler is a bioinformatics tool that integrates multiple functional analysis methods, featuring high efficiency in enrichment analysis and providing effective result visualization. The R package “clusterProfiler” was used for GO function enrichment analysis and KEGG pathway enrichment analysis (26). Statistical significance was set at adjusted *p* < 0.05. The PPI network was constructed using the STRING database (https://string-db.org/) with a confidence score > 0.4.

### Construction and validation of the prognostic model (GMGscore)

Using GSE65682 as the training set, LASSO (Least Absolute Shrinkage and Selection Operator)-Cox regression analysis was performed with the “glmnet” package (27). The optimal *λ* value was determined via 10-fold cross-validation to screen genes significantly associated with 28-day mortality in sepsis. The GMGscore for each patient was calculated based on the regression coefficients using the formula: GMGscore = ∑ (Expᵢ × Coefᵢ), where Coefᵢ represents the coefficient of the gene, and Expᵢ denotes the standardized expression level of that gene.

The “pROC” package (28) was used to generate receiver operating characteristic (ROC) curves, and the area under the curve (AUC) was calculated to evaluate the predictive performance of GMGscore for 28-day mortality in sepsis. Patients were divided into high-risk and low-risk groups according to the median GMGscore. Kaplan–Meier (KM) curves were plotted using the “survival” package, and the log-rank test was used to compare differences in survival rates between the two groups.

A nomogram was constructed by integrating GMGscore with clinical variables. The clinical utility of the nomogram was validated through ROC analysis, calibration curves, and decision curve analysis (DCA).

### Gene set enrichment analysis (GSEA) and immune infiltration analysis

GSEA was used to identify significantly altered key biological processes and pathways in the high-GMGscore group. The CIBERSORT algorithm (29) was applied to quantify the relative infiltration abundance of 22 immune cell types in the training set. Spearman correlation analysis was performed to explore the associations between key genes and immune cells.

### Screening of key targets and molecular docking

Based on information on key genes from the GutMGene database, Cytoscape software was used to construct a “gut microbiota-metabolite-host gene” interaction network. Targets meeting all the following criteria were screened: Significantly associated with sepsis prognosis (*p* < 0.05); Consistently differentially expressed in sepsis samples across all datasets; The regulatory direction of the gene by gut microbiota/metabolites is consistent with the direction of change that improves prognosis.

Structures of targets protein and gut microbiota metabolites were obtained from the UniProt database (https://www.uniprot.org/) and PubChem website (https://pubchem.ncbi.nlm.nih.gov/). Molecular docking was performed using AutoDock Vina (Version 1.2.0), with a binding energy < −5 kcal/mol as the screening criterion. The binding mode was visualized using PyMOL. AutoDock (https://ccsb.scripps.edu/mgltools/downloads/) and PyMOL software were used to simulate the binding between target proteins and small-molecule drugs.

### Blood sample collection

Blood samples were collected from sepsis patients (*n* = 6) and healthy donors (*n* = 6). The Modified Early Warning Score-Sepsis Recognition System (MEWS-SRS) was adopted for sepsis screening (30). Patients enrolled in this study received standardized care for sepsis in accordance with the guidelines established by the Surviving Sepsis Campaign (5). Informed consent was obtained from all participants who provided blood samples, and blood sample collection was approved by the Ethics Committee of Tongde Hospital of Zhejiang Province.

### RT-qPCR

Total RNA was extracted using TRIzol reagent (Thermo Fisher Scientific), with 1,000 ng of RNA serving as the template for first-strand cDNA synthesis. Real-time quantitative polymerase chain reaction (RT-qPCR) amplification was performed using the SYBR Green Supermix kit (Bio-Rad, Hercules, CA, USA). The relative expression levels of ASPM, CYP1A2, FFAR2, IL4R, MUC1, and RORA were determined by the 2^−^ΔΔCT method, with *β*-actin used as the internal reference gene. The primers used in this experiment are listed in Supplementary Table S3.

### Single-cell RNA sequencing (scRNA-seq) data analysis

Based on the scRNA-seq data from GSE167363, the expression distribution of key targets across different cell types was analyzed. Secondary dimensionality reduction and clustering were performed on cell types with high target expression to clarify the specific cell subpopulation localization of the targets.

The scTenifoldKnk method (31) was used to simulate target gene knockout and infer expression changes of downstream related genes. This method has been proven to be an efficient systematic tool for studying gene function, particularly suitable for research environments where real knockout (KO) experiments are not feasible. The single-cell dataset was normalized and preprocessed, and the scTenifoldKnk method was used to construct a gene regulatory network (GRN) that captures the regulatory relationships between genes in sepsis patients. In the constructed GRN, unsupervised virtual knockdown was performed by approximating the deletion of target gene nodes to identify genes with significantly altered regulatory relationships due to target gene knockdown. The significance of these genes was ranked based on Z-scores and *p*-values, and the expression change patterns of upregulated and downregulated genes were visualized.

### Statistical analysis

All statistical analyses were performed using R software (Version 4.3.1). For the comparison of continuous variables between two groups, an independent samples t-test or the Wilcoxon rank-sum test was used to analyze differences of variables. In Spearman correlation analysis, a correlation coefficient (R) > 0.3 was considered the correlation threshold. All statistical p-values were two-sided, and *p* < 0.05 was considered statistically significant. Statistical significance was denoted as follows: * indicates *p* < 0.05, ** indicates *p* < 0.01, *** indicates *p* < 0.001, and **** indicates *p* < 0.0001.

## Results

### Screening and functional characteristics of candidate genes

**Figure 2:**
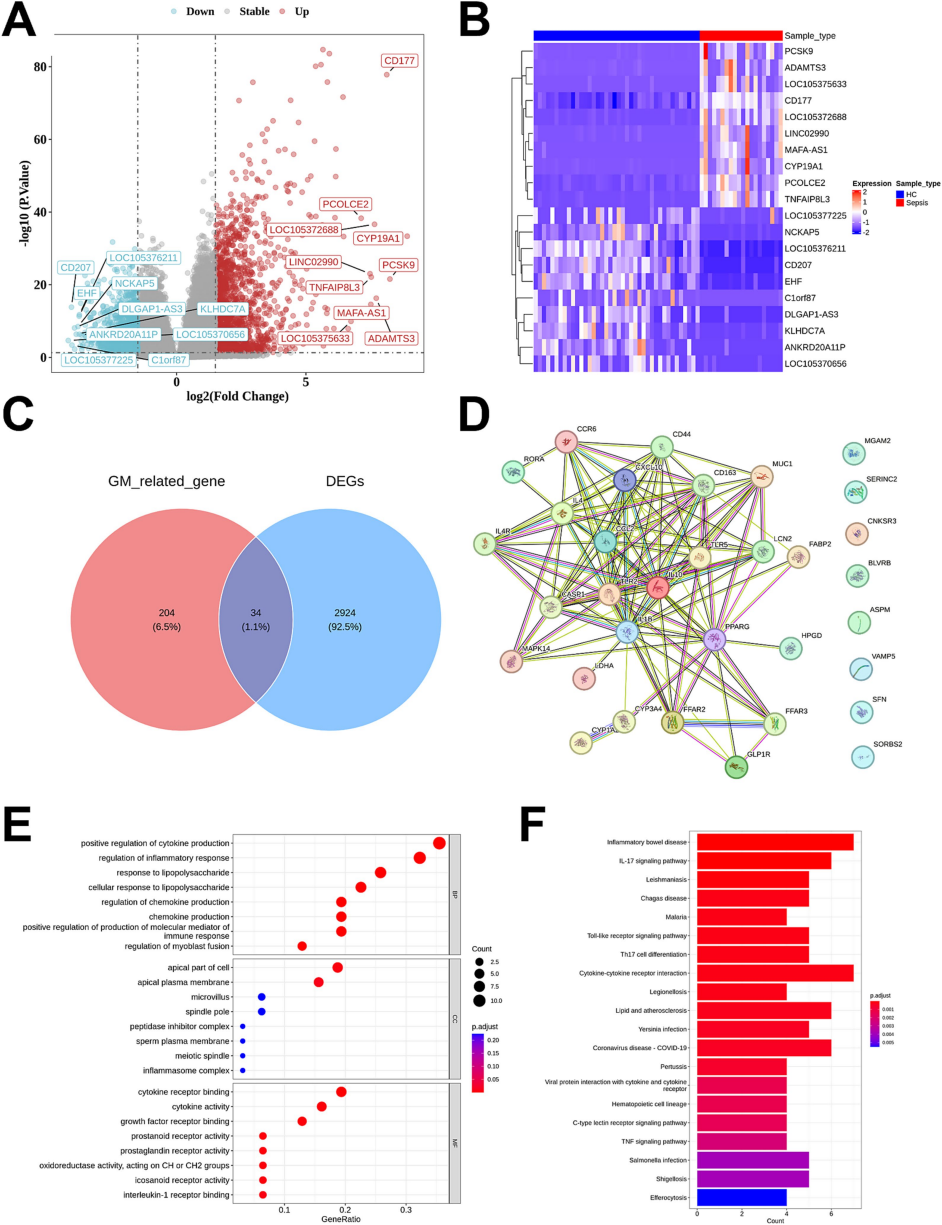
Identification of gut microbiota-related DEGs between sepsis and healthy control, followed by PPI, GO and KEGG analysis. **(A)** DEGs shown on a volcano plot in GSE154981. **(B)** Heatmap demonstration of Top 10 genes identified via differential expression analysis. **(C)** Venn diagram showed the intersected genes of gut microbiota-related genes and DEGs. **(D)** PPI network of 34 gut microbiota-related DEGs. **(E)** GO functional enrichment analysis of 34 gut microbiota-related DEGs. **(F)** KEGG pathway enrichment analysis of 34 gut microbiota-related DEGs.

In the GSE154918 dataset, a total of 2,958 DEGs were identified between sepsis patients and healthy controls, including 1,705 upregulated genes and 1,253 downregulated genes (Figure 2A). A heatmap was used to visualize the top 10 upregulated and downregulated genes ranked by fold change (Figure 2B). By taking the intersection of these DEGs with 238 gut microbiota-associated genes, 34 gut microbiota-related DEGs were obtained as candidate genes (Figure 2C). A PPI network diagram was constructed to illustrate the associations among these candidate genes (Figure 2D), and the chromosomal localization of these genes was visualized (Supplementary Figure S1).

GO analysis revealed significant enrichment in biological processes such as positive regulation of cytokine production, regulation of inflammatory response, response to lipopolysaccharide, and chemokine production. Additionally, enrichment was also observed in specific cellular components including the apical part of cell, apical plasma membrane, and inflammasome complex. Furthermore, enrichment was noted in molecular functions such as cytokine receptor binding, cytokine activity, and prostaglandin receptor activity (Figure 2E). The KEGG pathway enrichment analysis revealed that the candidate genes were mainly involved in pathways such as inflammatory bowel disease, IL-17 signaling pathway, and cytokine-cytokine receptor interaction (Figure 2F).

### Construction and validation of the prognostic model (GMGscore)

**Figure 3:**
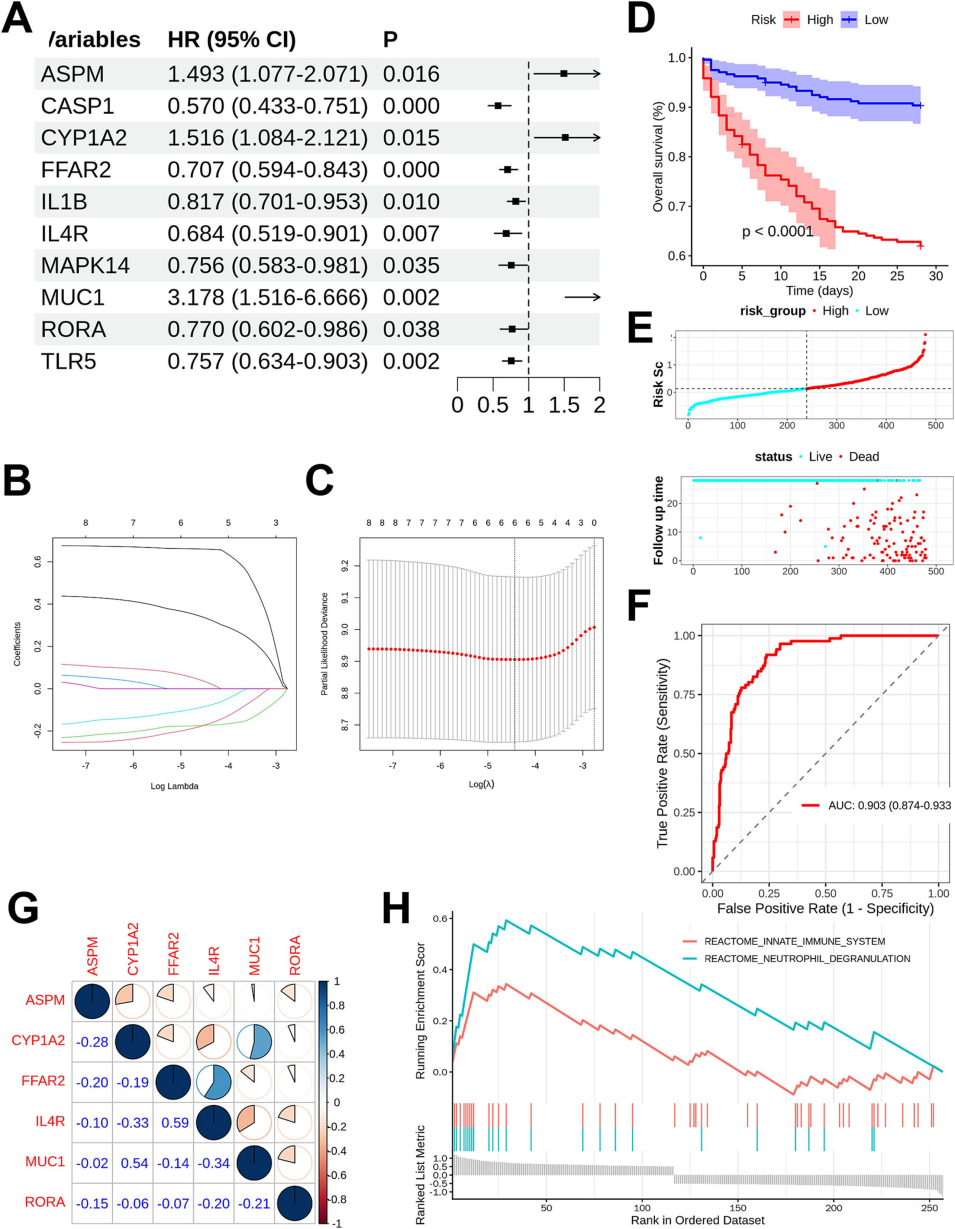
Construction and validation of the GMGscore. **(A)** Univariate Cox regression for identifying the prognosis-related genes in the gut microbiota-related DEGs. **(B)** LASSO coefficient path plot. **(C)** Cross-validation curve of LASSO regression analysis. **(D)** Survival analysis showed a significantly worse prognosis in the high-GMGscore group. **(E)** The distribution of GMGscore and outcome status. **(F)** The ROC curve of the training cohort demonstrated AUC value of 0.903. **(G)** Spearman correlation among the six prognosis genes. **(H)** GSEA results associated with GMGscore.

Using GSE65682 as the training set, univariate Cox analysis identified 10 genes significantly associated with prognosis (Figure 3A). Subsequently, LASSO regression analysis (*λ* = 0.01) was performed to screen 6 genes (CYP1A2, FFAR2, IL4R, MUC1, RORA, and ASPM) from the 10 candidate genes that were significantly correlated with 28-day mortality in sepsis patients, and the corresponding prognostic model was designated as GMGscore (Figures 3B,C). The genes included in GMGscore and their respective coefficients are provided in Supplementary Table S4.

Patients in the dataset were divided into high-risk and low-risk groups based on the median GMGscore. Kaplan–Meier (KM) analysis demonstrated that sepsis patients in the high-risk group had significantly poorer prognosis (*p* < 0.001) (Figure 3D). The GMGscore risk score-death event distribution plots (Figures 3E,F) and heatmap (Supplementary Figure S2) further confirmed that higher GMGscore was associated with worse prognosis. Additionally, subgroup survival analyses were conducted to verify the generalizability of GMGscore. The results showed that GMGscore exhibited robust prognostic discrimination ability across subgroups of male (Supplementary Figure S3A), female (Supplementary Figure S3B), elderly (Supplementary Figure S3C), non-elderly (Supplementary Figure S3D), diabetic (Supplementary Figure S3E), and non-diabetic (Supplementary Figure S3F) sepsis patients. ROC analysis indicated that GMGscore had excellent performance in prognostic prediction in the training set (AUC = 0.903, 95% confidence interval [CI]: 0.874–0.933) (Figure 3G). Validation using the GSE95233 dataset (Supplementary Figure S4) confirmed the strong generalization ability of GMGscore (AUC = 0.901, 95% CI: 0.813–0.990).

Spearman correlation analysis was performed to evaluate the expression correlations of the 6 key prognostic genes in sepsis samples (Figure 3H). Significant positive correlations were observed between FFAR2 and IL4R (*R* = 0.59), and between MUC1 and CYP1A2 (*R* = 0.54). In contrast, IL4R showed significant negative correlations with MUC1 (*R* = −0.34) and CYP1A2 (*R* = −0.33).

To explore the biological significance associated with GMGscore, GSEA was conducted. The results showed that the “innate immune system” [normalized enrichment score (NES) = 2.187, *p* = 0.001] and “neutrophil degranulation” (NES = 3.089, *p* < 0.001) were significantly upregulated in samples with high GMGscore. Furthermore, immune infiltration analysis revealed that sepsis patients in the high-GMGscore group had fewer neutrophils (*p* < 0.001) (Supplementary Figure S5). Spearman correlation test also confirmed a significant negative correlation between GMGscore and neutrophil levels in sepsis patients (*R* = −0.304, *p* < 0.001) (Supplementary Figure S6). In addition, compared with the low-GMGscore group, the high-GMGscore group had a reduction in activated mast cells and an increase in resting mast cells (*p* < 0.001).

### Association between GMGscore and clinical variables, and assessment of the nomogram

**Figure 4:**
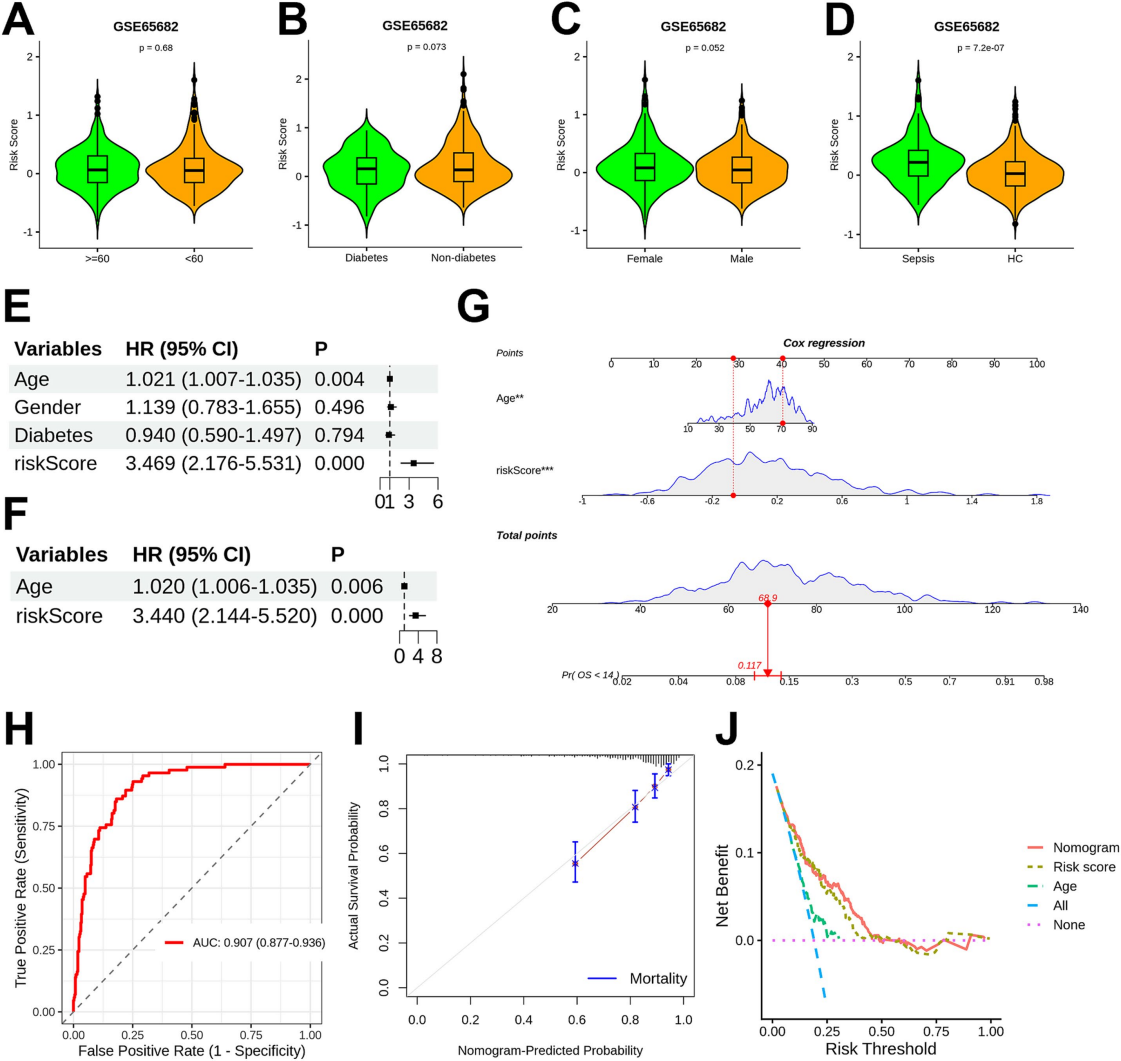
Association between GMGscore and linical variables, and the construction of a nomogram. **(A)** Violin plot showing that no significant difference was found between the GMGscores of sepsis patients younger than 60 years and those aged 60 years or older. **(B)** Violin plot illustrating no significant difference in GMGscores between diabetic and non-diabetic sepsis patients. **(C)** Violin plot demonstrating no significant difference in GMGscores between male and female sepsis patients. **(D)** Violin plot illustrating significant difference in GMGscores between healthy individuals and sepsis patients. **(E)** Univariate Cox regression for identifying the prognosis-related variables. **(F)** Multivariate Cox regression results of age and GMGscore. **(G)** A nomogram was developed via integrating clinical data and GMGscores. **(H)** ROC analysis for the nomogram showed the AUC value of 0.907. **(I)** Calibration curve for the nomogram. **(J)** DCA result demonstrated superior performance compared to single indicators.

GMGscore was compared across different subgroups to assess its association with clinical variables. The results showed no significant associations between GMGscore and age (Figure 4A; Supplementary Figure S7A), diabetes status (Figure 4B), or gender (Figure 4C; Supplementary Figures S7B,C). However, GMGscore was significantly higher in sepsis patients than in healthy controls across all datasets (Figure 4D; Supplementary Figures S7D–F).

Univariate and multivariate Cox regression analyses were performed by integrating clinical variables (Figures 4E,F). Based on these results, a nomogram was constructed using GMGscore and age (H*R* = 1.021, *p* = 0.002) as predictive parameters (Figure 4G). ROC analysis showed that the nomogram had an AUC of 0.907 (95% CI: 0.877–0.936) for prognostic prediction (Figure 4H). Calibration curves demonstrated the excellent prognostic performance of the nomogram (Figure 4I). DCA revealed that the nomogram provided greater clinical benefits than GMGscore or age alone (Figure 4J). Collectively, these results suggest that GMGscore has the potential to serve as a reliable clinical tool for sepsis prognosis prediction.

### Screening of key targets and molecular docking with potential therapeutic metabolites

**Figure 5:**
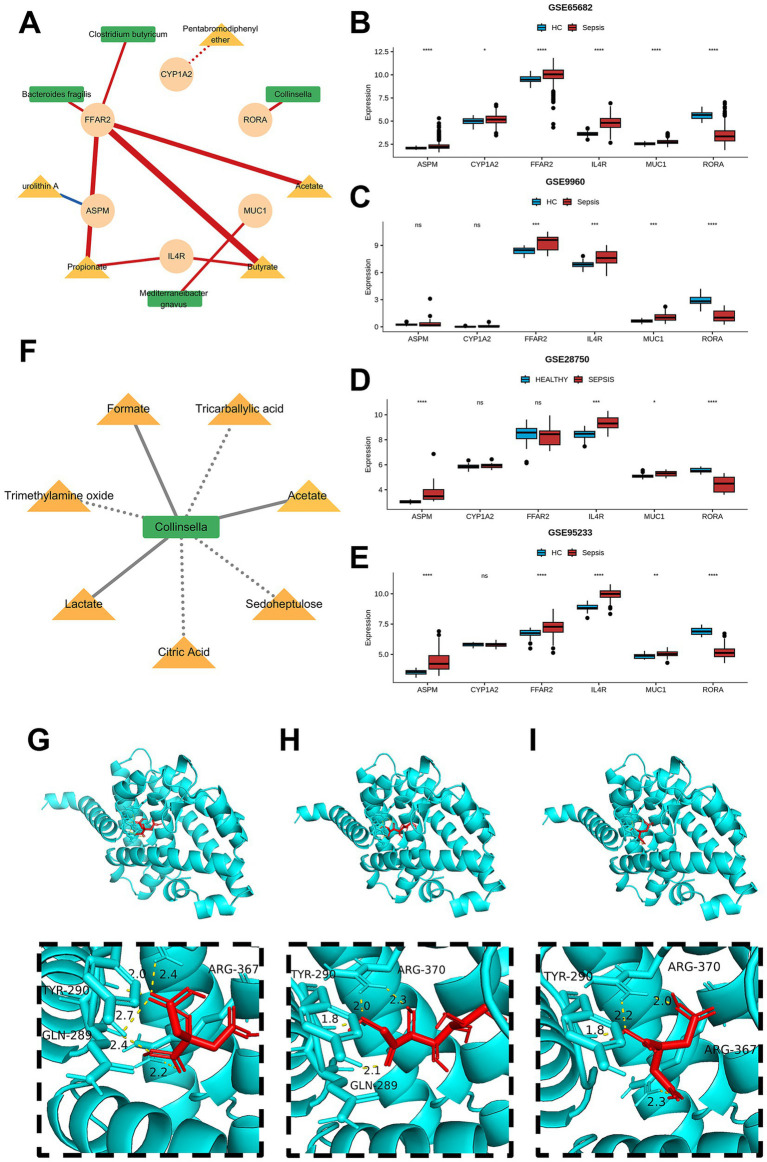
Screening of potential target for sepsis and promising metabolites for the treatment. **(A)** Network for demonstrating the relationship among six prognosis genes and microbiota/metabolites. **(B)** Boxplot showing the expression level of six prognosis genes in GSE65682. **(C)** Boxplot showing the expression level of six prognosis genes in GSE9960. **(D)** Boxplot showing the expression level of six prognosis genes in GSE28750. **(E)** Boxplot showing the expression level of six prognosis genes in GSE95233. **(G)** Molecular docking result of RORA and citric acid. **(H)** Molecular docking result of RORA and sedoheptulose. **(I)** Molecular docking result of RORA and tricarballylic acid.

We visualized the regulatory relationships between prognostic genes and gut microbiota/metabolites (Figure 5A). In the visualization, red lines represent activation, blue lines represent inhibition, dashed lines represent correlation, and solid lines represent causal association. Notably, only urolithin A exerted an inhibitory effect on ASPM.

We further verified the consistency of prognostic gene expression across five datasets: GSE65682, GSE95233, GSE28750, GSE9960. The results showed that IL4R and MUC1 were consistently highly expressed in sepsis patients, while Retinoic Acid Receptor-Related Orphan Receptor Alpha (RORA) was consistently lowly expressed (*p* < 0.001, Figures 5B–D). We also obtained RNA-seq data from an Asian septic cohort (GSE243219) and found that RORA was also significantly downregulated in the sepsis group (*p* < 0.001, Supplementary Figure S8). Additionally, RORA exhibited the highest AUC value for sepsis diagnosis across all five datasets (Supplementary Figure S9A–E). In the interaction network, we observed that Collinsella could activate RORA, which aligns with the direction of improved sepsis prognosis (Figure 5A). Therefore, we consider RORA a potential target that can be intervened via the gut microbiota.

Collinsella was found to have correlational relationships with 7 metabolites: Acetate, Citric acid, Formate, Lactate, Sedoheptulose, Tricarballylic acid, and Trimethylamine oxide (Figure 5E). Molecular docking results (Supplementary Table S5) revealed that three of these metabolites formed relatively stable bindings with RORA protein: Citric acid (binding energy = −5.5 kcal/mol), Sedoheptulose (binding energy = −5.2 kcal/mol), Tricarballylic acid (binding energy = −5.6 kcal/mol). Notably, the predicted binding sites of all three metabolites included the tyrosine residue at position 290 (Figures 5F–H).

### Validation of six genes expression in human samples

**Figure 6:**
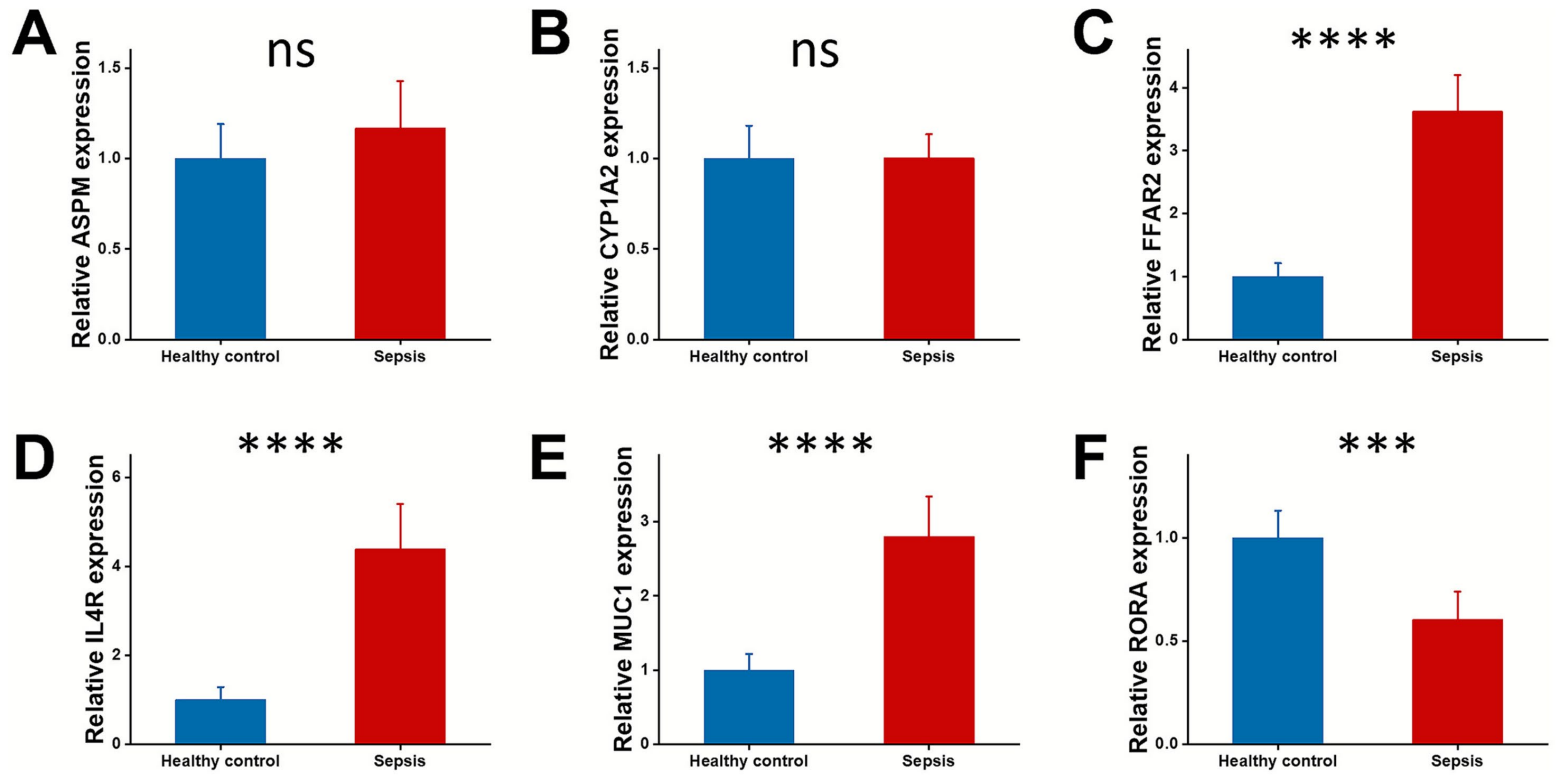
Expression of the six prognosis genes in sepsis patients. Blood samples were collected from sepsis patients (*n* = 6) and normal control (*n* = 6) and examined for the mRNA expression levels of ASPM **(A)**, CYP1A2 **(B)**, FFAR2 **(C)**, IL4R **(D)**, MUC1 **(E)**, and RORA **(F)** using RT-qPCR.

To further verify the role of these prognostic genes, we collected blood samples from sepsis patients and healthy controls, and measured the mRNA expression levels of ASPM, CYP1A2, FFAR2, IL4R, MUC1, and RORA. As shown in Figure 6, compared with healthy controls, there were no statistically significant differences in the mRNA expression levels of ASPM and CYP1A2 in the blood samples of sepsis patients (Figures 6A,B). In contrast, the mRNA expression levels of FFAR2, IL4R, and MUC1 were significantly upregulated in sepsis patients (Figures 6C-E), while the mRNA expression level of RORA was significantly downregulated (Figure 6F).

### Single-cell RNA-seq data analysis

**Figure 7:**
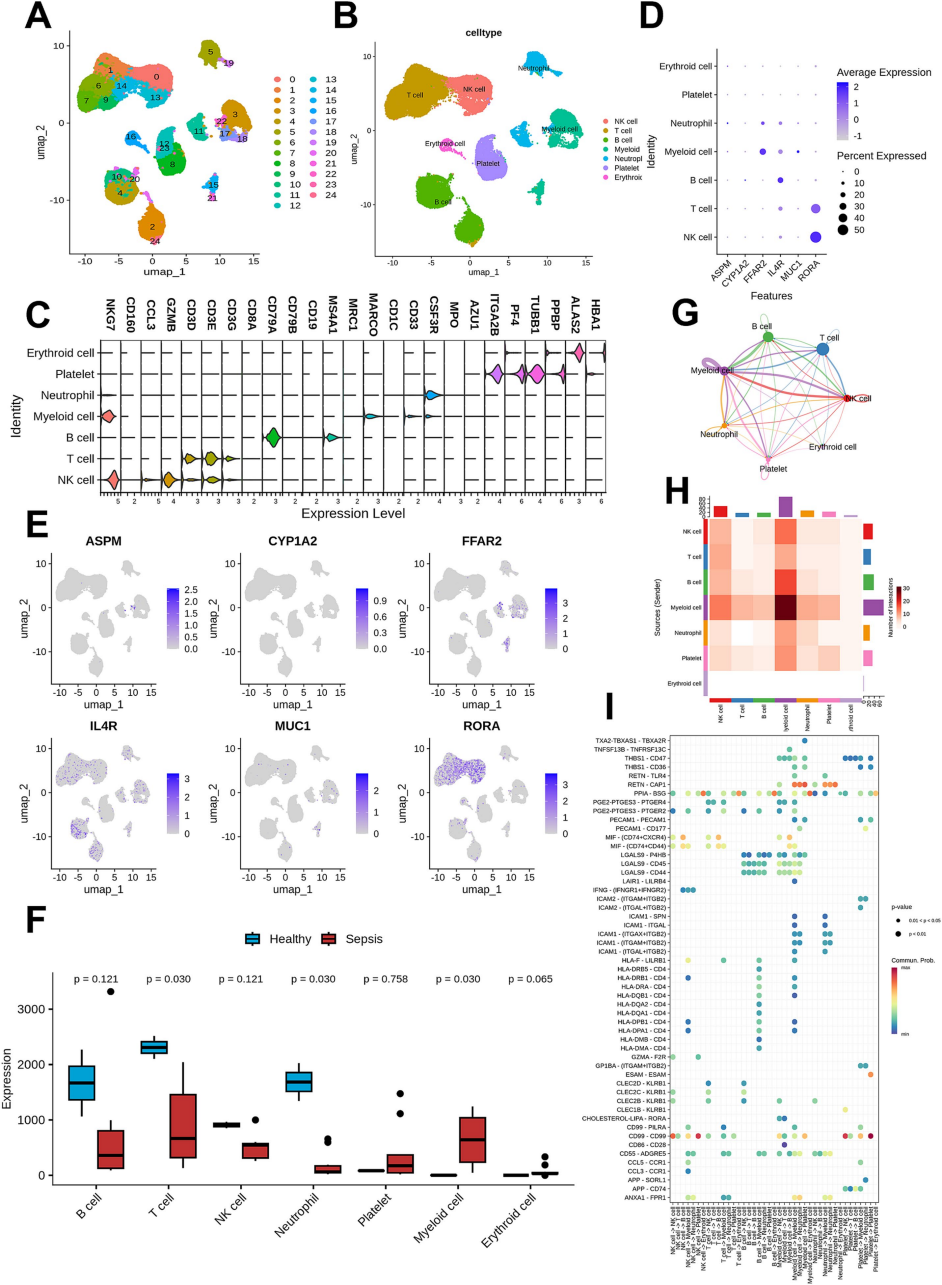
Single-cell RNA sequencing data analysis. **(A)** A total of 25 clusters were identified using UMAP clustering. **(B)** Cell types of the dataset. **(C)** Marker genes of different cell clusters. **(D)** Dot plot showing the expression levels of the six prognosis genes in different cell types. **(E)** UMAP plot demonstrating the cell types distribution of the six prognosis genes. **(F)** Comparison for the cell counts of different cell types between sepsis and healthy control. **(G)** Network plot result of cell communication analysis. **(H)** Heatmap result of cell communication analysis. **(I)** Bubble plot showing the ligand-receptor pair of cell–cell interaction.

The scRNA-seq data (GSE167363) used in this study included 12 samples in total. After data preprocessing (filtering low-quality cells with mitochondrial gene ratio >10% and UMI count <200), a total of 33,525 high-quality cells were retained for subsequent analysis (Supplementary Figure S10). Using the UMAP method, all cells were clustered into 25 clusters and annotated into 7 cell types: NK cells, T cells, B cells, neutrophils, myeloid cells, platelets, and erythroid cells (Figure 6B). The differential expression of marker genes across different cell types confirmed the reliability of cell clustering (Figure 7D).

We then mapped the distribution of prognostic genes in these cells (Figures 7D,E), and the analysis showed that RORA was mainly expressed in T cells and NK cells. By comparing the counts of various cell types between healthy samples and sepsis samples, we found that T cells, neutrophils and myeloid cells were significantly reduced in sepsis samples; thus, we focused on T cells in subsequent analyses.

CellChat was used to visualize the intercellular communication network in sepsis samples (Figures 6G,H), which showed that myeloid cells had a higher communication probability with other cell types. Additionally, the ligand-receptor pairs mediating intercellular communication in sepsis were identified and presented (Figure 7I).

**Figure 8:**
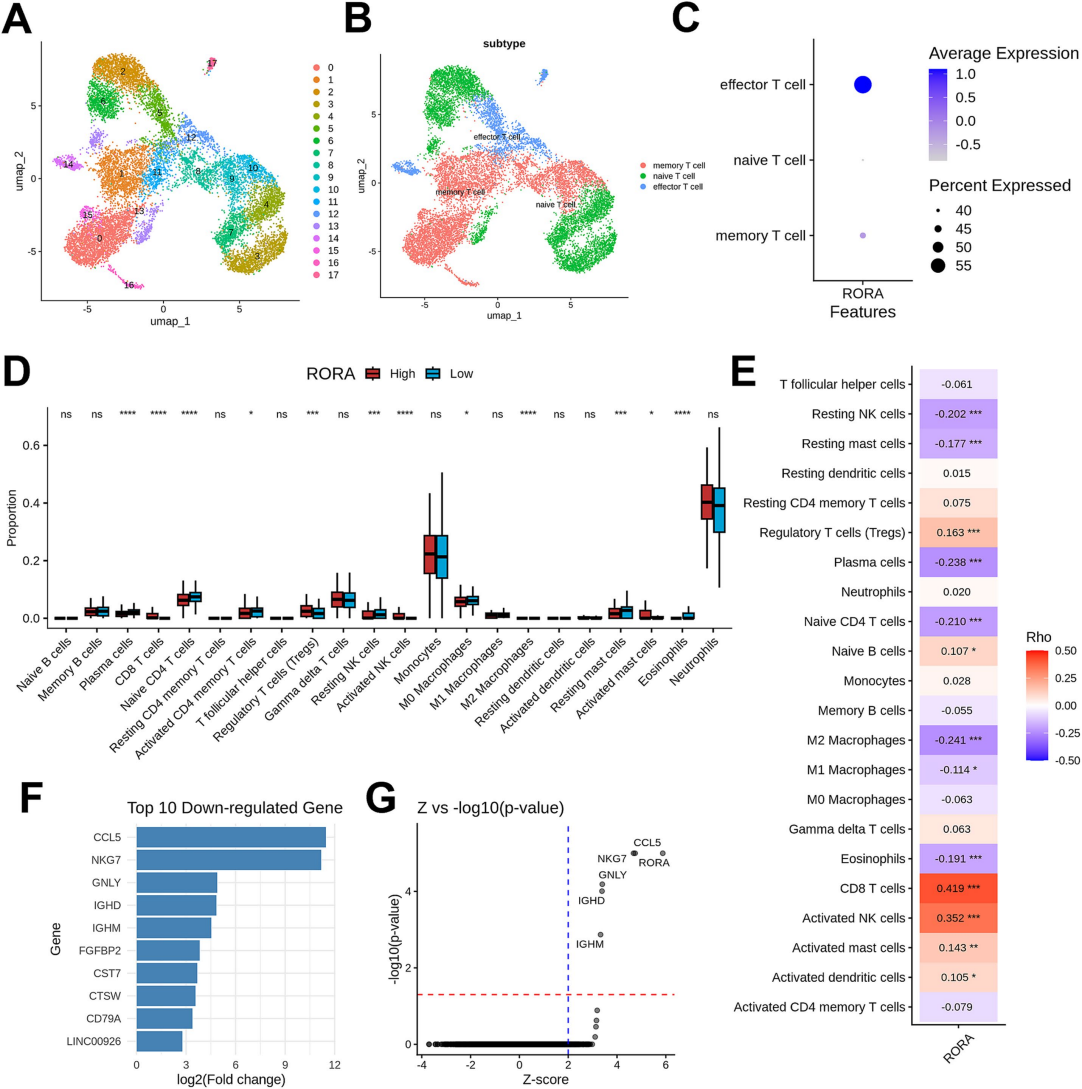
The role of RORA in sepsis was inferred through multiple analytical methods. **(A)** A total of 18 clusters were identified from T cell subset using UMAP clustering. **(B)** T cell subtypes of the subset. **(C)** Dot plot showing the RORA expression level among different T cell subtypes. **(D)** Immune infiltration analysis associated with RORA expression. **(E)** Spearman correlation between RORA and 22 types of immune cells. **(F)** Bar plot showing the top 10 down-regulated genes after virtual KO of RORA. **(G)** Volcano plot showing significantly dysregulated genes following virtual KO of RORA.

To further explore T cells, we subclassified the focused T cells into subsets (Figures 8A,B). The results indicated that RORA was primarily distributed in effector T cells (Figure 8C). Immune infiltration analysis (Figure 8D) revealed that samples with high RORA expression had higher immune infiltration levels of CD8 + T cells and activated NK cells, while the infiltration levels of naive CD4 + T cells and resting NK cells were lower.

Spearman correlation analysis (Figure 8E; Supplementary Figures S11A,B) demonstrated that RORA was significantly positively correlated with the levels of CD8 + T cells (*R* = 0.419, *p* < 0.001) and activated NK cells (*R* = 0.352, *p* < 0.001).

We also conducted virtual knockout analysis (Figures 8F,G). After virtual knockout of RORA, the expression levels of genes such as CCL5, NKG7, and GNLY were significantly downregulated. This suggests that RORA may influence the immune balance in sepsis by regulating the function or recruitment of effector T cells and NK cells.

## Discussion

Risk stratification of sepsis serves as a critical basis for early targeted intervention, and gut microbiota-associated biomarkers offer a more comprehensive perspective for prognostic assessment in critical care medicine (19). Against the backdrop of the big data era, multi-omics data has provided robust support for the prediction of sepsis prognosis (32).

Through integrated multi-omics analysis, this study constructed, for the first time, a sepsis prognostic model (GMGscore) based on gut microbiota-related genes. The model demonstrated excellent performance in both the training set and validation set, with AUC values reaching 0.903 and 0.901, respectively. It is expected to be applied in clinical practice to identify high-risk sepsis patients, thereby enabling early targeted treatment. For comparison, the 28-day mortality prediction model based on autophagy-related genes developed by Chen et al. had an AUC of 0.700 (33), while their HLA-related classifier showed an AUC range of 0.691–0.752 (34). Jiang et al. established a prognostic model using an inflammatory response-related gene signature, which achieved an AUC of 0.866 (35), and the prognostic model constructed by Jiang et al. had an AUC range from 0.707 to 0.856 (36). These results further underscore the significance of gut microbiota in sepsis prognosis.

We explored the correlations of GMGscore with clinical features, immune infiltration, and pathway enrichment. In the analysis of clinical correlations, GMGscore showed no association with age, gender, or the presence of diabetes, indicating that GMGscore is a relatively independent prognostic indicator. Consistently, GMGscore was elevated in sepsis patients. Immune infiltration analysis revealed that the number of neutrophils decreased with increasing GMGscore (*R* = −0.304, *p* < 0.001), and scRNA seq analysis showed a similar result (*p* = 0.030). A reduction in neutrophil count may imply impaired anti-infective capacity, thereby contributing to the progression of sepsis (37). GSEA pathway enrichment analysis showed upregulation of pathways such as neutrophil degranulation and the innate immune system. The activation of neutrophil degranulation and the innate immune system are hallmark upregulated pathways in early sepsis, which may indicate excessive activation of these biological processes in high-risk sepsis patients (38). Excessive activation of neutrophil degranulation, while effective in killing pathogens, can also damage the normal tissues and organs of the organism (39). Notably, neutrophil dysfunction is a core pathological event in sepsis (40). These findings suggest that the high-risk status defined by GMGscore may be mediated through changes in neutrophil quantity and function.

The genus Collinsella is typically described as a strictly anaerobic pathogenic bacterium that produces lactic acid rather than butyrate or other short-chain fatty acids (41). It is closely associated with non-alcoholic steatohepatitis and cholesterol metabolism and is considered a pro-inflammatory pathogenic genus (42, 43). However, the results of our study suggested that Collinsella may improve sepsis prognosis by activating RORA through its metabolites.

This study also identified RORA as a potential target amenable to gut microbiota-based intervention. RORA is a transcription factor belonging to the nuclear receptor superfamily, and it plays roles in multiple processes including neural function, cell development, immune regulation, metabolism, and circadian rhythm (44). Previous studies have confirmed that RORA is involved in inflammatory diseases by regulating the circadian clock and immune cell activation (45). Upregulation of RORA can prevent inflammation and inhibit the expression of adhesion-related proteins, including intercellular adhesion molecule 1 (ICAM-1) and vascular cell adhesion molecule 1 (VCAM-1) in human umbilical vein endothelial cells (HUVECs) (46).

RORA expression is downregulated in sepsis patients and is regarded as a key regulator (47, 48). Previous studies have shown that RORA exhibits high diagnostic performance for sepsis (49), which is consistent with the findings of our study. Experiments in mice have confirmed that RORA is a key factor in initiating the innate immune response against inflammation and exerts a protective role during the inflammatory process (50). The present study found that RORA is lowly expressed in sepsis patients and is significantly associated with poor prognosis. Besides, RORA is mainly enriched in effector T cells and NK cells. Additionally, gut microbiota metabolites including citric acid, sedoheptulose, and tricarballylic acid may bind to RORA, suggesting that gut microbiota may regulate the immune response in sepsis patients through the metabolite-RORA interaction.

In our study, the significant positive correlation between RORA and CD8 + T cells/NK cells highlights its positive role in regulating immune cell function. Furthermore, results from virtual knockdown of RORA showed that low RORA expression downregulates the expression of cytotoxic genes such as CCL5, NKG7, and GNLY. This may restrict the recruitment and functional exertion of T cells and NK cells, thereby mediating immune suppression (51, 52). In animal experiments, upregulation of RORA expression using sevoflurane alleviated lipopolysaccharide-induced endothelial cell damage (53). Targeting RORA to restore circadian rhythm may represent an innovative therapeutic approach to alleviate immune dysfunction and improve patient prognosis (54).

This study has certain limitations. First, it relies on public datasets, and the samples are mainly derived from European populations. Future study should include more data from a broader range of Asian populations to further validate the generalizability of the GMGscore. Second, the sample size of scRNA-seq data is relatively small, so the cellular localization results of RORA need to be verified with an expanded sample size. Third, the study is limited to bioinformatics analysis; cell and animal experiments are required to validate the regulatory role of RORA expression and activity on the body’s immune system, as well as its impact on sepsis-related inflammatory factors. Future studies should focus on validating the interactions between the small molecules identified in this study and the RORA protein, conducting functional validation experiments of RORA in immune cell models, and assessing therapeutic efficacy in animal models of sepsis. Finally, this study did not include clinical samples for protein-level validation. Future multi-center clinical studies are needed to verify the clinical utility of GMGscore and RORA-targeted interventions.

## Conclusion

Through integrated analysis of transcriptome and single-cell sequencing data, this study constructed a sepsis prognostic model (GMGscore) based on 6 gut microbiota-related genes. This model exhibits excellent prognostic discrimination ability and holds potential clinical application value. Meanwhile, RORA was identified as a potential target for gut microbiota-based intervention. It may influence the immune balance in sepsis by regulating the functions of effector T cells and NK cells. These findings not only deepen the understanding of the mechanism by which gut microbiota contributes to sepsis progression but also provide a novel perspective for the precise prognostic assessment and targeted treatment of sepsis. Multi-center clinical validation and functional experiments are required to further promote the clinical translational application of GMGscore and RORA.

## Data Availability

The datasets presented in this study can be found in online repositories. The names of the repository/repositories and accession number(s) can be found in the article/Supplementary material.

## Glossary

AUC: Area under the curve
BP: Biological Process
CC: Cellular Component
DCA: Decision curve analysis
DEG: Differentially expressed gene
GEO: Gene Expression Omnibus
GMGscore: Gut microbiota related genes score
GO: Gene Ontology
GRN: Gene regulatory network
GSEA: Gene Set Enrichment Analysis
HUVEC: Human umbilical vein endothelial cell
ICAM-1: Intercellular adhesion molecule 1
ICU: intensive care units
KEGG: Kyoto Encyclopedia of Genes and Genomes
KM: Kaplan–Meier
KO: Knockout
MF: Molecular Function
PPI: Protein–Protein Interaction
RORA: Retinoic Acid Receptor-Related Orphan Receptor Alpha
scRNA-seq: Single-Cell RNA Sequencing
UMAP: Uniform Manifold Approximation and Projection
UMI: Unique molecular identifier
VCAM-1: vascular cell adhesion molecule 1

## References

[1] Gavelli F Castello LM Avanzi GC. Management of sepsis and septic shock in the emergency department. Intern Emerg Med. (2021) 16:1649–61. doi: 10.1007/s11739-021-02735-7, 33890208 PMC8354945

[2] Rudd KE Johnson SC Agesa KM Shackelford KA Tsoi D Kievlan DR . Global, regional, and national sepsis incidence and mortality, 1990-2017: analysis for the global burden of disease study. Lancet. (2020) 395:200–11. doi: 10.1016/s0140-6736(19)32989-7, 31954465 PMC6970225

[3] Torio CM Moore BJ. National Inpatient Hospital Costs: The Most Expensive Conditions by Payer, 2013. Healthcare Cost and Utilization Project (HCUP) Statistical Briefs. Rockville (MD): Agency for Healthcare Research and Quality (US) (2013). 27359025

[4] McBride MA Patil TK Bohannon JK Hernandez A Sherwood ER Patil NK. Immune checkpoints: novel therapeutic targets to attenuate Sepsis-induced immunosuppression. Front Immunol. (2020) 11:624272. doi: 10.3389/fimmu.2020.624272, 33613563 PMC7886986

[5] Rhodes A Evans LE Alhazzani W Levy MM Antonelli M Ferrer R . Surviving Sepsis campaign: international guidelines for Management of Sepsis and Septic Shock: 2016. Intensive Care Med. (2017) 43:304–77. doi: 10.1007/s00134-017-4683-6, 28101605

[6] Naime ACA Ganaes JOF Lopes-Pires ME. Sepsis: the involvement of platelets and the current treatments. Curr Mol Pharmacol. (2018) 11:261–9. doi: 10.2174/1874467211666180619124531, 29921214

[7] Henriquez-Camacho C Losa J. Biomarkers for sepsis. Biomed Res Int. (2014) 2014:547818. doi: 10.1155/2014/547818, 24800240 PMC3985161

[8] Sweeney TE Wong HR. Risk stratification and prognosis in Sepsis: what have we learned from microarrays? Clin Chest Med. (2016) 37:209–18. doi: 10.1016/j.ccm.2016.01.003, 27229638 PMC4884300

[9] Barichello T Generoso JS Singer M Dal-Pizzol F. Biomarkers for sepsis: more than just fever and leukocytosis-a narrative review. Crit Care. (2022) 26:14. doi: 10.1186/s13054-021-03862-5, 34991675 PMC8740483

[10] Li W Zheng C Zhang X Wang B Shen E Wang L . Stimulation of soluble guanylyl cyclase (sGC) by Cinaciguat attenuates Sepsisinduced cardiac injury. Curr Mol Pharmacol. (2024) 17:e18761429387280. doi: 10.2174/0118761429387280250506114040, 40396316

[11] Wong KF Luk JM. Endotoxin-neutralizing peptides as gram-negative sepsis therapeutics. Protein Pept Lett. (2009) 16:539–42. doi: 10.2174/092986609788167761, 19442233

[12] Chen WY Wang M Zhang J Barve SS McClain CJ Joshi-Barve S. Acrolein disrupts tight junction proteins and causes endoplasmic reticulum stress-mediated epithelial cell death leading to intestinal barrier dysfunction and permeability. Am J Pathol. (2017) 187:2686–97. doi: 10.1016/j.ajpath.2017.08.015, 28935573 PMC5818631

[13] Assimakopoulos SF Triantos C Thomopoulos K Fligou F Maroulis I Marangos M . Gut-origin sepsis in the critically ill patient: pathophysiology and treatment. Infection. (2018) 46:751–60. doi: 10.1007/s15010-018-1178-5, 30003491

[14] Miller WD Keskey R Alverdy JC. Sepsis and the microbiome: a vicious cycle. J Infect Dis. (2021) 223:S264–s269. doi: 10.1093/infdis/jiaa682, 33330900 PMC8206800

[15] Tauber SC Nau R. Immunomodulatory properties of antibiotics. Curr Mol Pharmacol. (2008) 1:68–79. 20021425

[16] Schuijt TJ Lankelma JM Scicluna BP de Sousa e Melo F Roelofs JJ de Boer JD . The gut microbiota plays a protective role in the host defence against pneumococcal pneumonia. Gut. (2016) 65:575–83. doi: 10.1136/gutjnl-2015-309728, 26511795 PMC4819612

[17] Fay KT Klingensmith NJ Chen CW Zhang W Sun Y Morrow KN . The gut microbiome alters immunophenotype and survival from sepsis. FASEB J. (2019) 33:11258–69. doi: 10.1096/fj.201802188R, 31306584 PMC6766641

[18] Ma X Jia X Peng Y Li X Wang C Yu K. Gut microbiota disruption during sepsis and the influence of innate metabolites on sepsis prognosis. International Microbiol. (2023) 26:929–38. doi: 10.1007/s10123-023-00349-x, 36967434

[19] Wei R Chen X Hu L He Z Ouyang X Liang S . Dysbiosis of intestinal microbiota in critically ill patients and risk of in-hospital mortality. Am J Transl Res. (2021) 13:1548–57. 33841678 PMC8014420

[20] Kim SM DeFazio JR Hyoju SK Sangani K Keskey R Krezalek MA . Fecal microbiota transplant rescues mice from human pathogen mediated sepsis by restoring systemic immunity. Nat Commun. (2020) 11:2354. doi: 10.1038/s41467-020-15545-w, 32393794 PMC7214422

[21] Assimakopoulos SF Papadopoulou I Bantouna D de Lastic AL Rodi M Mouzaki A . Fecal microbiota transplantation and hydrocortisone ameliorate intestinal barrier dysfunction and improve survival in a rat model of Cecal ligation and puncture-induced Sepsis. Shock (Augusta, Ga). (2021) 55:666–75. doi: 10.1097/shk.0000000000001566, 32496421

[22] Qi C He G Qian K Guan S Li Z Liang S . gutMGene v2.0: an updated comprehensive database for target genes of gut microbes and microbial metabolites. Nucleic Acids Res. (2025) 53:D783–d788. doi: 10.1093/nar/gkae1002, 39475181 PMC11701569

[23] Butler A Hoffman P Smibert P Papalexi E Satija R. Integrating single-cell transcriptomic data across different conditions, technologies, and species. Nat Biotechnol. (2018) 36:411–20. doi: 10.1038/nbt.4096, 29608179 PMC6700744

[24] Aran D Looney AP Liu L Wu E Fong V Hsu A . Reference-based analysis of lung single-cell sequencing reveals a transitional profibrotic macrophage. Nat Immunol. (2019) 20:163–72. doi: 10.1038/s41590-018-0276-y, 30643263 PMC6340744

[25] Love MI Huber W Anders S. Moderated estimation of fold change and dispersion for RNA-seq data with DESeq2. Genome Biol. (2014) 15:550. doi: 10.1186/s13059-014-0550-8, 25516281 PMC4302049

[26] Wu T Hu E Xu S Chen M Guo P Dai Z . clusterProfiler 4.0: a universal enrichment tool for interpreting omics data. Innovation. (2021) 2:100141. doi: 10.1016/j.xinn.2021.100141, 34557778 PMC8454663

[27] Tay JK Narasimhan B Hastie T. Elastic net regularization paths for all generalized linear models. J Stat Softw. (2023) 106:1–24. doi: 10.18637/jss.v106.i01, 37138589 PMC10153598

[28] Robin X Turck N Hainard A Tiberti N Lisacek F Sanchez JC . pROC: an open-source package for R and S+ to analyze and compare ROC curves. BMC Bioinformatics. (2011) 12:77. doi: 10.1186/1471-2105-12-77, 21414208 PMC3068975

[29] Newman AM Liu CL Green MR Gentles AJ Feng W Xu Y . Robust enumeration of cell subsets from tissue expression profiles. Nat Methods. (2015) 12:453–7. doi: 10.1038/nmeth.3337, 25822800 PMC4739640

[30] Croft CA Moore FA Efron PA Marker PS Gabrielli A Westhoff LS . Computer versus paper system for recognition and management of sepsis in surgical intensive care. J Trauma Acute Care Surg. (2014) 76:311–7. doi: 10.1097/ta.0000000000000121, 24458039

[31] Osorio D Zhong Y Li G Xu Q Yang Y Tian Y . scTenifoldKnk: an efficient virtual knockout tool for gene function predictions via single-cell gene regulatory network perturbation. Patterns. (2022) 3:100434. doi: 10.1016/j.patter.2022.100434, 35510185 PMC9058914

[32] Spottiswoode N Neyton LP Mick E Kalantar KL Hao S Lydon EC . Host-microbe Multiomic profiling predicts mortality in Sepsis. Am J Respir Crit Care Med. (2025). doi: 10.1164/rccm.202410-1996OC, 40788839 PMC12927001

[33] Chen Z Zeng L Liu G Ou Y Lu C Yang B . Construction of autophagy-related gene classifier for early diagnosis, prognosis and predicting immune microenvironment features in Sepsis by machine learning algorithms. J Inflamm Res. (2022) 15:6165–86. doi: 10.2147/jir.S386714, 36386585 PMC9653048

[34] Chen Z Chen R Ou Y Lu J Jiang Q Liu G . Construction of an HLA classifier for early diagnosis, prognosis, and recognition of immunosuppression in Sepsis by multiple transcriptome datasets. Front Physiol. (2022) 13:870657. doi: 10.3389/fphys.2022.870657, 35685286 PMC9171028

[35] Jiang S Zhang W Lu Y. Development and validation of novel inflammatory response-related gene signature for sepsis prognosis. J Zhejiang Univ Sci B. (2022) 23:1028–41. doi: 10.1631/jzus.B2200285, 36518055 PMC9758714

[36] Jiang J Chen Y Su Y Zhang L Qian H Song X . Identification and experimental validation of diagnostic and prognostic genes CX3CR1, PID1 and PTGDS in sepsis and ARDS using bulk and single-cell transcriptomic analysis and machine learning. Front Immunol. (2024) 15:1480542. doi: 10.3389/fimmu.2024.1480542, 39763654 PMC11700820

[37] Wei Y Kim J Ernits H Remick D. The septic neutrophil-friend or foe. Shock. (2021) 55:147–55. doi: 10.1097/SHK.0000000000001620, 32769816

[38] Taha S Bindayna K Aljishi M Sultan A Almansour N. Transcriptomic profiling reveals distinct immune dysregulation in early-stage sepsis patients. Int J Mol Sci. (2025) 26:6647. doi: 10.3390/ijms26146647, 40724897 PMC12294327

[39] Magallon M Castillo-Corullon S Banuls L Romero T Pellicer D Herrejon A . Impact of hypoxia on neutrophil degranulation and inflammatory response in Alpha-1 antitrypsin deficiency patients. Antioxidants. (2024) 13:1071. doi: 10.3390/antiox13091071, 39334730 PMC11428696

[40] Martin-Fernandez M Tamayo-Velasco A Aller R Gonzalo-Benito H Martinez-Paz P Tamayo E. Endothelial dysfunction and neutrophil degranulation as central events in sepsis physiopathology. Int J Mol Sci. (2021) 22:6272. doi: 10.3390/ijms22126272, 34200950 PMC8230689

[41] Chen J Wright K Davis JM Jeraldo P Marietta EV Murray J . An expansion of rare lineage intestinal microbes characterizes rheumatoid arthritis. Genome Med. (2016) 8:43. doi: 10.1186/s13073-016-0299-7, 27102666 PMC4840970

[42] Gomez-Arango LF Barrett HL Wilkinson SA Callaway LK McIntyre HD Morrison M . Low dietary fiber intake increases Collinsella abundance in the gut microbiota of overweight and obese pregnant women. Gut Microbes. (2018) 9:189–201. doi: 10.1080/19490976.2017.1406584, 29144833 PMC6219589

[43] Astbury S Atallah E Vijay A Aithal GP Grove JI Valdes AM. Lower gut microbiome diversity and higher abundance of proinflammatory genus Collinsella are associated with biopsy-proven nonalcoholic steatohepatitis. Gut Microbes. (2020) 11:569–80. doi: 10.1080/19490976.2019.1681861, 31696774 PMC7524262

[44] Zang M Zhao Y Gao L Zhong F Qin Z Tong R . The circadian nuclear receptor RORalpha negatively regulates cerebral ischemia-reperfusion injury and mediates the neuroprotective effects of melatonin. Biochim Biophys Acta Mol basis Dis. (2020) 1866:165890. doi: 10.1016/j.bbadis.2020.165890 32599143

[45] Hams E Roberts J Bermingham R Fallon PG. Functions for retinoic acid-related orphan receptor alpha (RORα) in the activation of macrophages during lipopolysaccharide-induced septic shock. Front Immunol. (2021) 12:647329. doi: 10.3389/fimmu.2021.647329, 33767712 PMC7986717

[46] Huang H Liu X Chen D Lu Y Li J Du F . Melatonin prevents endothelial dysfunction in SLE by activating the nuclear receptor retinoic acid-related orphan receptor-α. Int Immunopharmacol. (2020) 83:106365. doi: 10.1016/j.intimp.2020.106365 32172204

[47] Oliveira RAC Imparato DO Fernandes VGS Cavalcante JVF Albanus RD Dalmolin RJS. Reverse engineering of the Pediatric Sepsis regulatory network and identification of master regulators. Biomedicine. (2021) 9:1297. doi: 10.3390/biomedicines9101297, 34680414 PMC8533457

[48] Chen YJ Lu JJ Lin CP Hu WC. Microarray analysis reveals Sepsis is a syndrome with hyperactivity of TH17 immunity, with over-presentation of the Treg cell cytokine TGF-β. Curr Issues Mol Biol. (2025) 47:435. doi: 10.3390/cimb47060435, 40699834 PMC12191643

[49] Zeng N Jian Z Xu J Peng T Hong G Xiao F. Identification of qualitative characteristics of immunosuppression in sepsis based on immune-related genes and immune infiltration features. Heliyon. (2024) 10:e29007. doi: 10.1016/j.heliyon.2024.e29007, 38628767 PMC11019180

[50] Stapleton CM Jaradat M Dixon D Kang HS Kim SC Liao G . Enhanced susceptibility of staggerer (RORalphasg/sg) mice to lipopolysaccharide-induced lung inflammation. Am J Physiol Lung Cell Mol Physiol. (2005) 289:L144–52. doi: 10.1152/ajplung.00348.2004, 15778248

[51] Roberts J Chevalier A Hawerkamp HC Yeow A Matarazzo L Schwartz C . Retinoic acid-related orphan receptor alpha is required for generation of Th2 cells in type 2 pulmonary inflammation. J Immunol. (2023) 211:626–32. doi: 10.4049/jimmunol.2200896, 37387671 PMC10404816

[52] Liu D Wei B Liang L Sheng Y Sun S Sun X . The circadian clock component RORA increases immunosurveillance in melanoma by inhibiting PD-L1 expression. Cancer Res. (2024) 84:2265–81. doi: 10.1158/0008-5472.CAN-23-3942, 38718296 PMC11247325

[53] Ni W Zou Z Jiang P Wang S. Sevoflurane alleviates inflammation, apoptosis and permeability damage of human umbilical vein endothelial cells induced by lipopolysaccharide by inhibiting endoplasmic reticulum stress via upregulating RORα. Prostaglandins Other Lipid Mediat. (2024) 172:106821. doi: 10.1016/j.prostaglandins.2024.106821, 38373554

[54] Teng X Wang Q Ma J Li D. Integrating bioinformatics and machine learning to discover sumoylation associated signatures in sepsis. Sci Rep. (2025) 15:14398. doi: 10.1038/s41598-025-96956-x, 40274894 PMC12022290

