# Supplementary material for: Development of a prognostic model for sepsis based on gut microbiota-associated genes and identification of potential targets
Source: Front Med (Lausanne). 2026 Mar 5;13:1766359. doi: 10.3389/fmed.2026.1766359 (PMC12999399; doi:10.3389/fmed.2026.1766359)
Supplement: Supplementary file 2 [file Supplementary_file_1.docx]

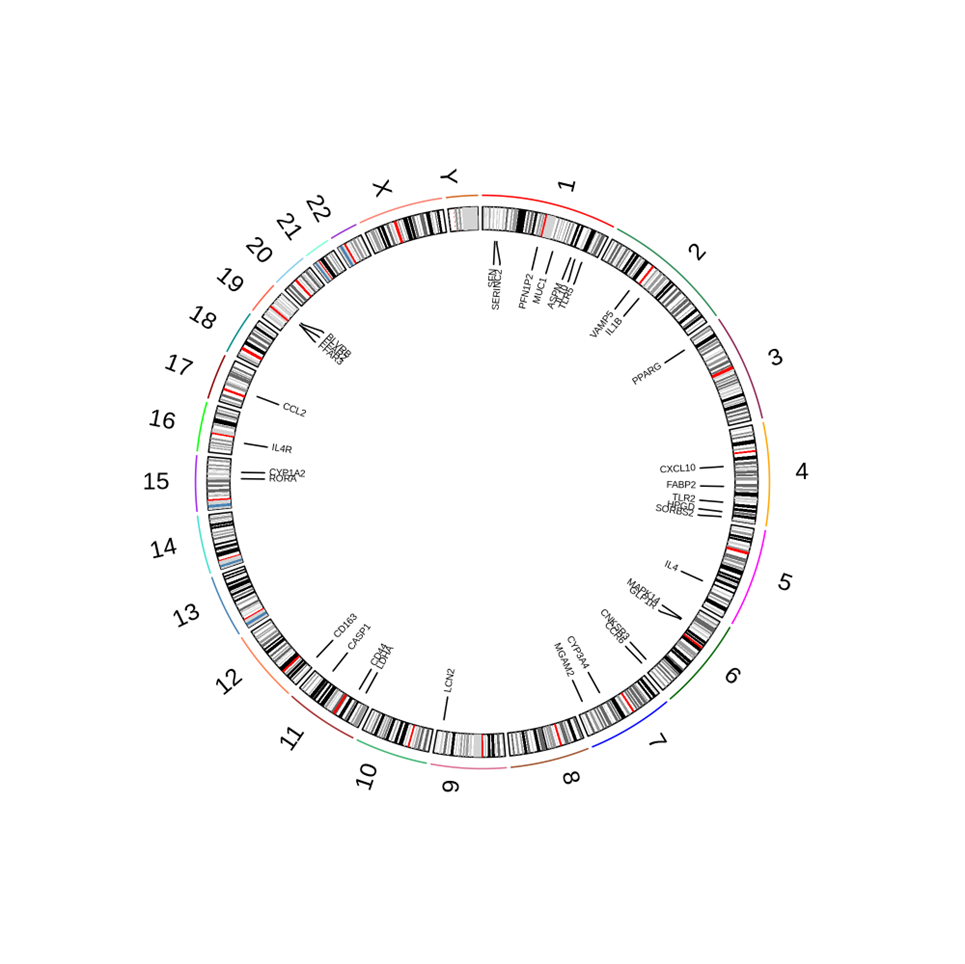


Fig. S1. Circle diagram showing gut microbiota-related DEGs at different positions on the chromosome.


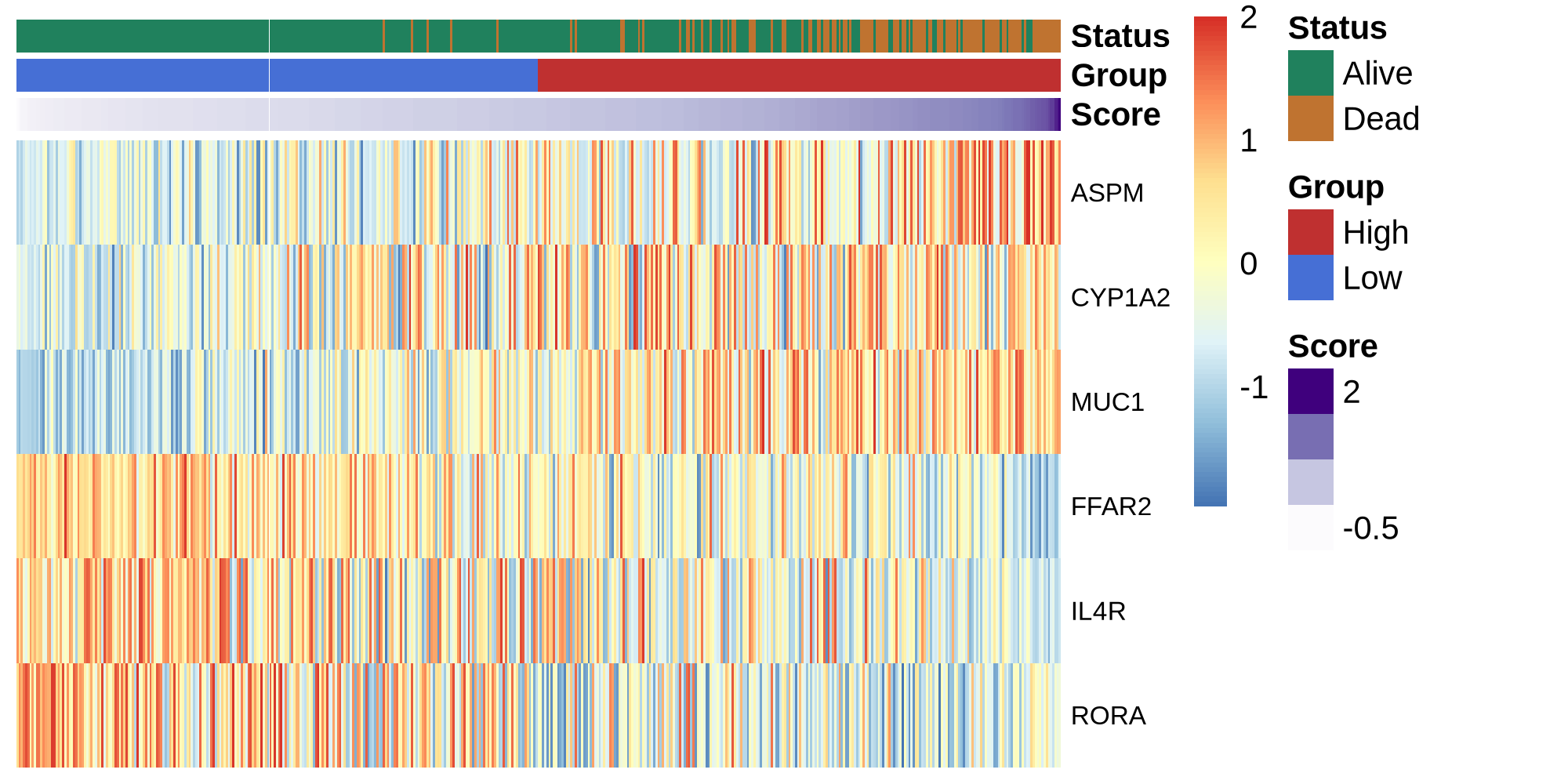


Fig. S2. Heatmap demonstrating the association among six prognosis genes expression, GMGscore, and survival status.


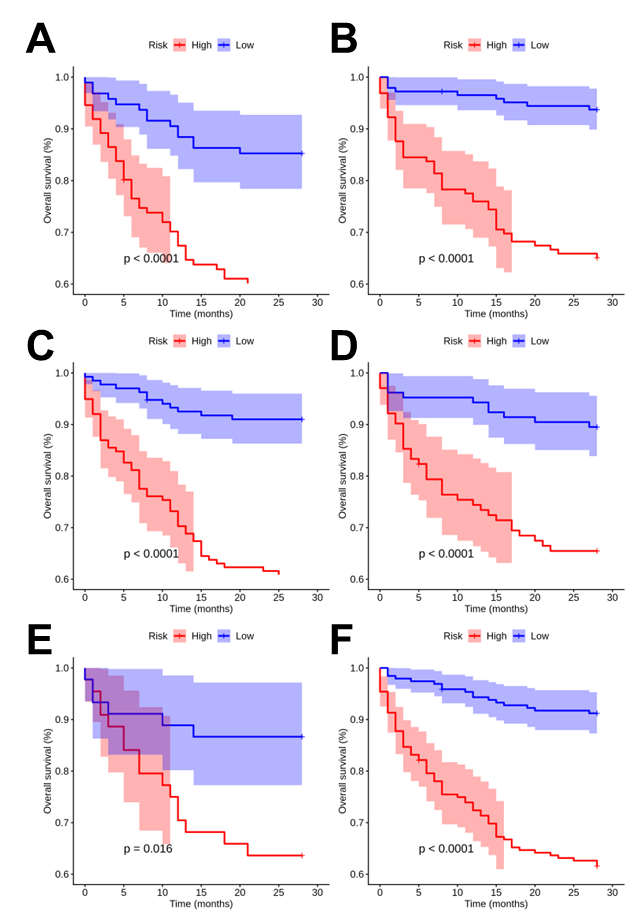


Fig. S3. Subgroup survival analysis in male (A), female (B), older than 60 (C), younger than 60 (D), diabetes (E), and non-diabetes (F) sepsis patients.


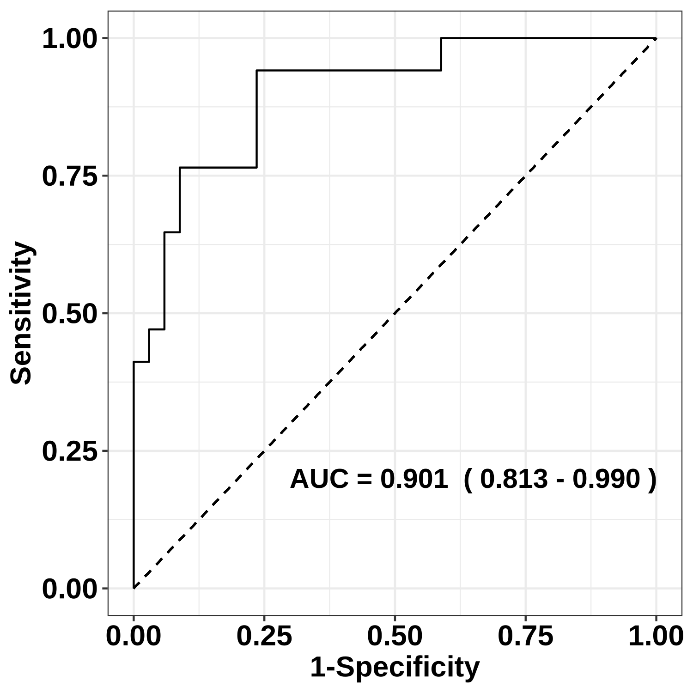


Fig. S4. ROC analysis showing the AUC values for validation set is 0.901.


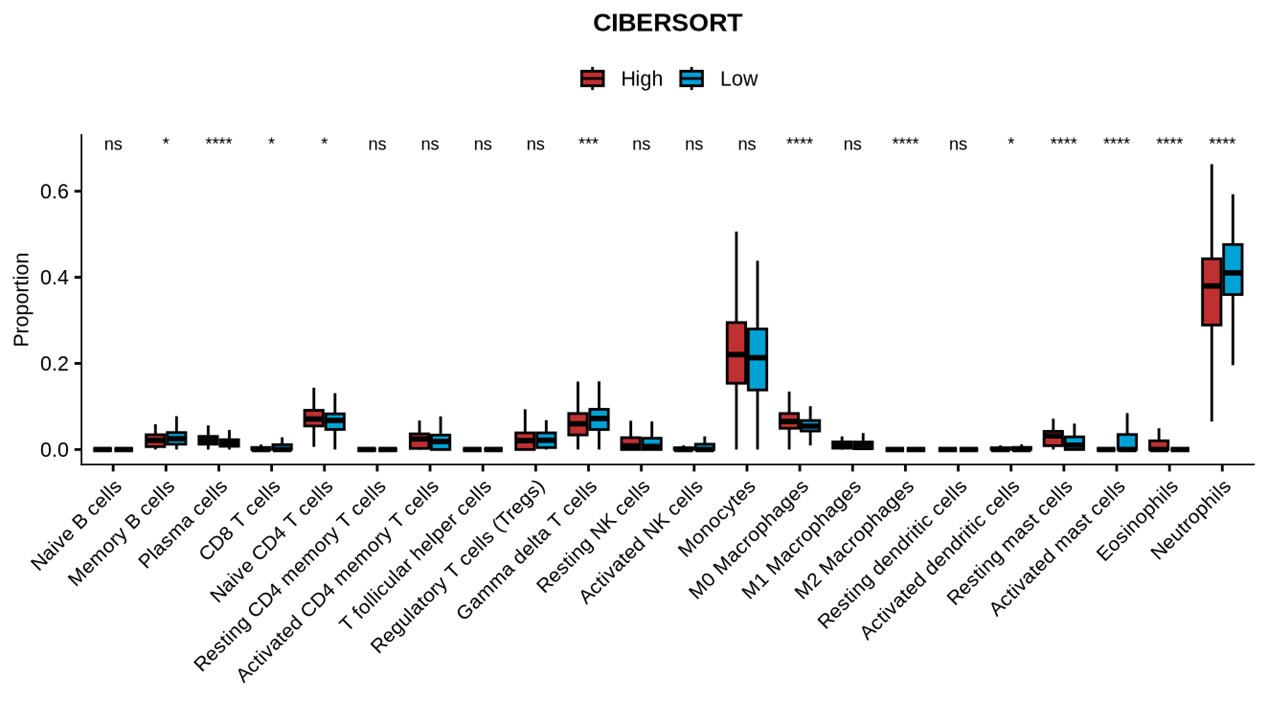


Fig. S5. CIBERSORT analysis for immune infiltration comparison between GMGscore-high group and GMG-score-low group.


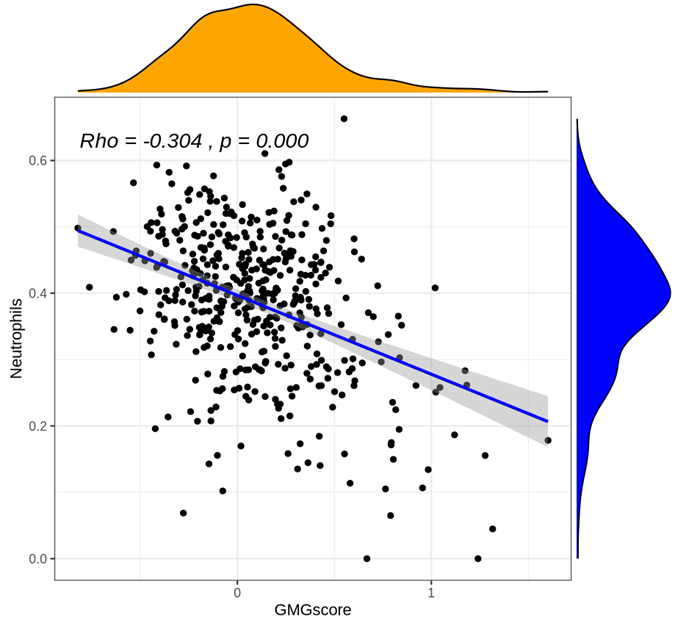


Fig. S6. Spearman correlation between GMGscore and neutrophils.


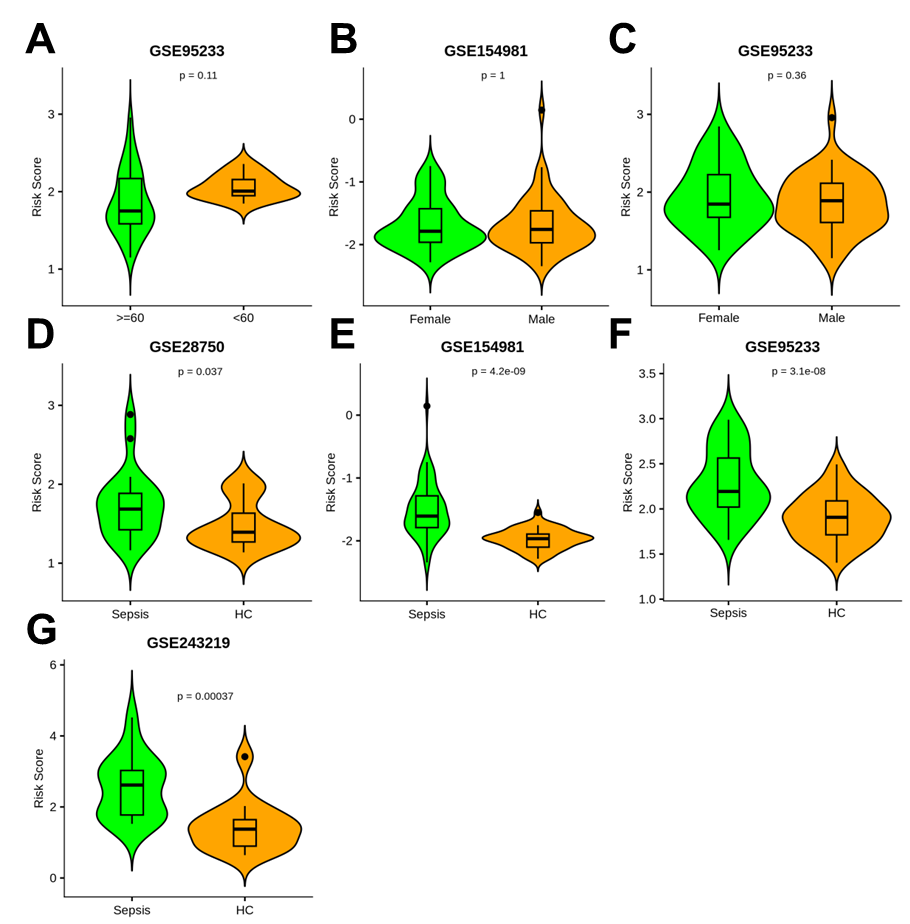


Fig. S7. Associations between clinical variables and GMGscore.


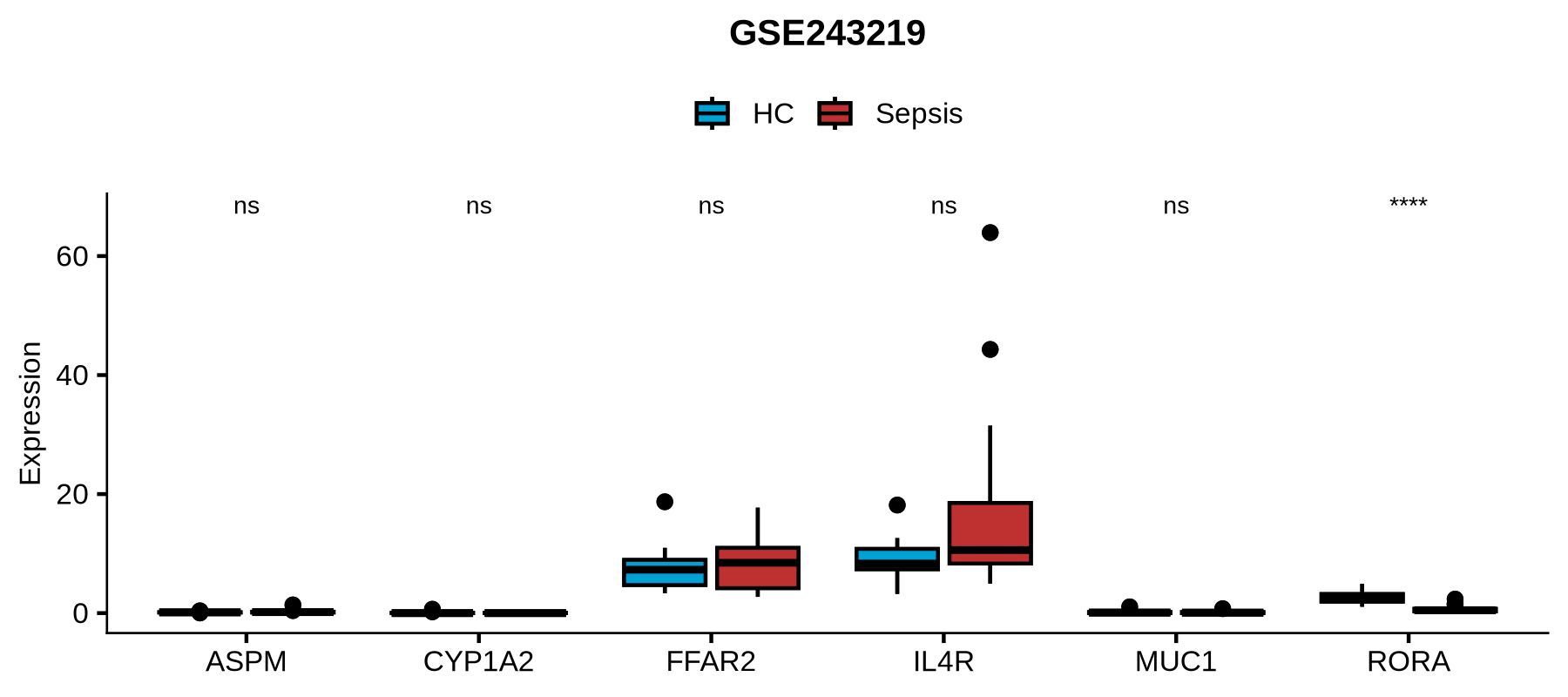


Fig. S8. Boxplot showing the expression level of six prognosis genes in GSE243219.


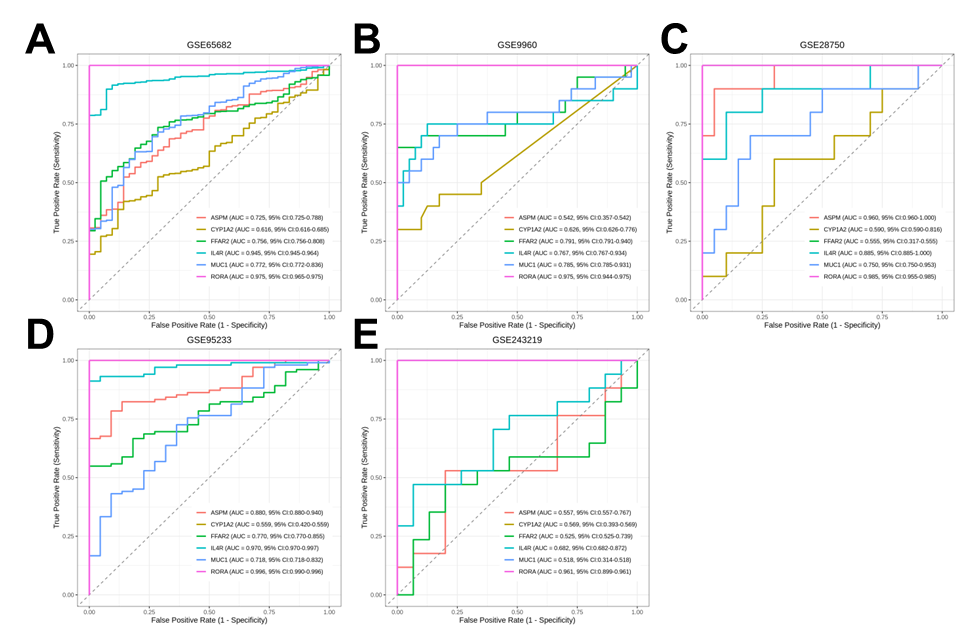


Fig. S9. ROC analysis of six prognosis genes for sepsis diagnosis in different dataset: (A) GSE65682; (B) GSE9960; (C) GSE28750; (D) GSE95233; (E) GSE243219.


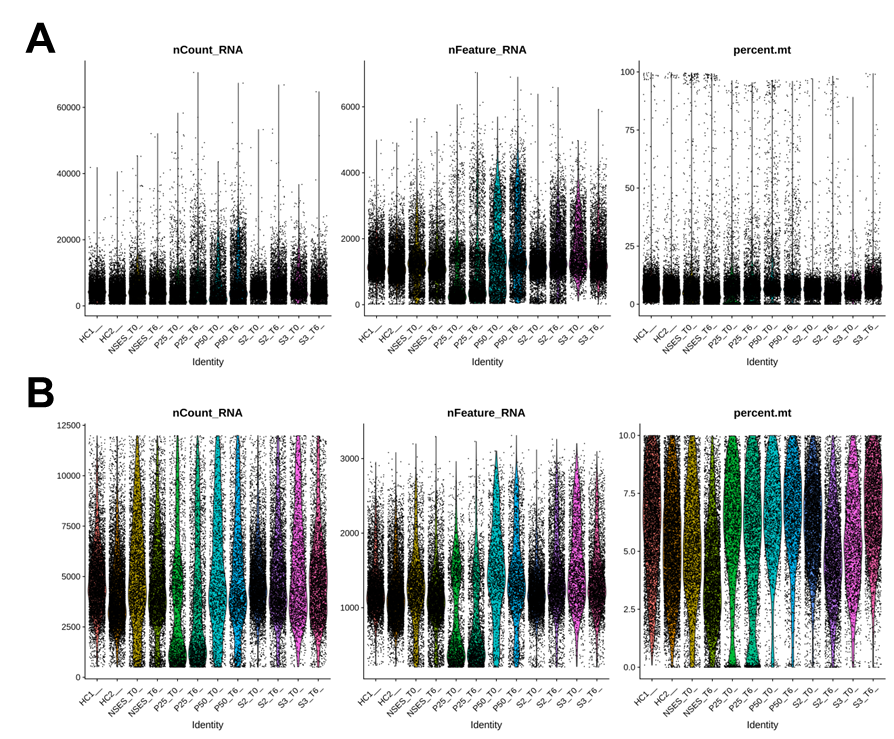


Fig. S10. The quality control of single cell data. (A) Violin plots depicting the RNA characteristic count (nFeature RNA), the absolute UMI count (nCount RNA), and the percent of mitochondrial genes (percent.mt) prior to cell quality control measures. (B) Violin plots showcasing the nFeature RNA, the nCount RNA, and the percent.mt post quality control filtering.


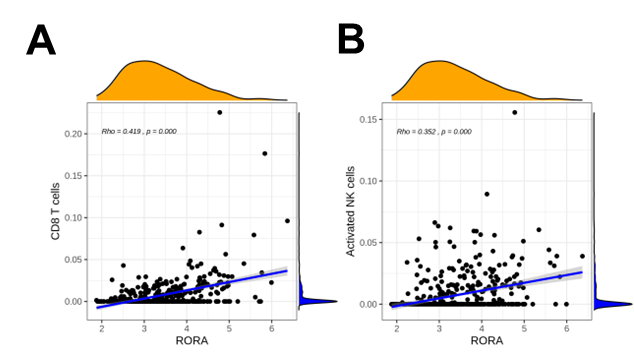


Fig. S11. Spearman correlation between RORA and CD8+ T cells (A)/NK cells (B) levels.
